# No apparent gain from continuing migration for more than 3000 kilometres: willow warblers breeding in Denmark winter across the entire northern Savannah as revealed by geolocators

**DOI:** 10.1186/s40462-017-0109-x

**Published:** 2017-08-30

**Authors:** Mathilde Lerche-Jørgensen, Mikkel Willemoes, Anders P. Tøttrup, Katherine Rachel Scotchburn Snell, Kasper Thorup

**Affiliations:** Center for Macroecology, Evolution and Climate, Natural History Museum of Denmark, University of Copenhagen, Universitetsparken 15, 2100 Copenhagen, Denmark

**Keywords:** Migration, Geolocation, Connectivity, Itinerancy

## Abstract

**Background:**

For most Afro-Palearctic migrants, particularly small songbirds, spatiotemporal migration schedules and migratory connectivity remain poorly understood. We mapped migration from breeding through winter of one of the smallest Afro-Palearctic migrants, the willow warbler *Phylloscopus trochilus*, using geolocators (*n* = 15).

**Results:**

Birds migrated from North European breeding grounds to West Africa via the Iberian Peninsula following a narrow corridor along the West Coast of Africa. Birds then dispersed across the northern Savannah with termination of migration highly variable among individuals. The termination of migration appeared not to be related to timing, current and previous years’ vegetation conditions or biometrics. During winter, most birds moved southwards to improved vegetation.

**Conclusion:**

The willow warblers showed a large, unexpected longitudinal spread in winter sites of more than 3000 km between individuals breeding within a 500 m range resulting in a low degree of connectivity. The large wintering area may well be related to generalist behaviour in the species. Our findings contribute to understanding the link between breeding and wintering ecology in long-distance migratory birds.

**Electronic supplementary material:**

The online version of this article (10.1186/s40462-017-0109-x) contains supplementary material, which is available to authorized users.

## Background

Migration is an adaptation to seasonal environments [1, 2] and billions of small songbirds travel vast distances from Palearctic breeding to sub-Saharan wintering grounds every year [3]. Understanding the drivers of these travels and the links to the seasonally changing environments requires detailed knowledge of the spatiotemporal migration pattern. Yet, for most of these species, our knowledge of migration routes, non-breeding stop-overs and wintering sites is incomplete. Gaining knowledge and understanding of these patterns is crucial in the light of their general declines [4, 5] and if we are to forecast potential effects of climate change [6].

Migratory connectivity, how much individuals from the same breeding site mix during the non-breeding season with birds from other sites within the same population [7], is essential for understanding ecology and population dynamics [8–11]. Connectivity is defined as a relative measure: It is high if little mixing occurs, i.e. individuals from different breeding sites stay separated in winter, and it can be low even if wintering occurs within a limited area as long as the whole population winters within the same area. Thus, connectivity can be inferred to be low if birds from a single site spread out over a large part of the known wintering area. Connectivity is commonly low in long-distance migratory birds [12] but some notable exceptions have been reported [9, 11].

Many European bird species wintering in West Africa follow a southwestern migration route into Africa [13, 14]. In West Africa, migration along the coast apparently occurs in several species, including passerines [11, 15, 16], only turning inland after reaching the Sahel [17–19]. Circumventing the Sahara along the coast is a relatively short route [20] and potentially allows a safe Sahara crossing with opportunities for foraging, drinking and predator avoidance. However, Biebach [21] concluded that the coast was unlikely to be a major flyway for most passerines because no mass migration had been observed.

Individuals of several species move between sedentary periods in Africa (itinerancy [22]). Itinerancy is thought to be a response to changing food conditions though direct links such as in raptors [23] are rarely documented. Tracking has revealed both sedentary behaviour [11, 15, 24, 25] and itinerant behaviour [26, 27] in species wintering in West and Central Africa.

The insectivorous willow warbler *Phylloscopus trochilus* is among the smallest of the Afro-Palearctic migrants. It breeds in a variety of woody habitats across the Palearctic and winters in the entire Savannah zone covering most of sub-Saharan Africa [28]. However, there is a considerable variation among subspecies and presumably a high degree of connectivity [29, 30] with ring recoveries supporting partial separation of subspecies also in winter [31]. The subspecies *P.* *t. trochilus* occurs in West Europe and southern East Europe, wintering in West Africa. Little is known about winter movements and both residency [32] and itinerancy [33] have been suggested.

Despite importance for understanding links to the seasonal environments as well as conservation [34], information about connectivity, migration routes and within-winter itinerancy [22] is generally scarce or lacking. Furthermore, few studies have evaluated the causes of connectivity at the behavioural level, for example migration direction or termination of migration [35]. Among-individual spread is often assumed to result from variation in migration directions [36] but several other factors could also be involved. Here, we use geolocators to describe migration routes, stopovers and wintering sites of male willow warblers *P.* *t. trochilus* breeding in Denmark. Despite the low spatial resolution, estimation of timing of movements and mapping of stopping-over locations at a regional scale is possible [37, 38]. We focus on the resulting migratory connectivity and the behaviours causing it at the individual level. Overall, we find surprisingly little connectivity (large longitudinal spread compared to the wintering distribution of the studied subspecies) and test a range of possible causes, such as filling up of suitable habitat, dominance hierarchy, and vegetation conditions. Furthermore, we describe itinerancy (movement between sedentary stays) and explore the link with environmental conditions. We use arrival data as a proxy for the potential filling up of suitable habitat. A potential dominance hierarchy is explored based on measurements of weight and wing length. To investigate effects of vegetation and environmental conditions, we explore patterns of the Normalized Difference Vegetation Index (NDVI) based on the assumption that vegetation greenness is ultimately related to food availability though the link is not direct [6].

## Methods

We fitted male willow warblers with Intigeo–W30 geolocators (Migrate Technology LTD, 0.3 g, *c.* seven months capacity) in East Denmark (55.61°N, 12.57°E; catching range 500 m) from May to mid-June (*n* = 17 in 2014, *n* = 20 in 2015) using leg-loop harnesses [39] made of 1 mm braided nylon cord. 17 birds were recaptured the year after tagging (*n* = 11 in 2015, *n* = 6 in 2016). Two loggers from 2014 contained no data.

Positions were estimated using the GeoLight package [40] in R [41]. A threshold of 3 lx was used and sun elevation angles between −3° and 0° provided the best fit using Hill-Ekström calibration [38] (breeding area calibration produced similar spatiotemporal patterns, Additional file 1: Appendix S2-S4).

Periods of no overall change in longitude for ≥5 days were considered staging. We excluded latitude from positions within ten days of equinox. Position outliers >10° from median longitude/latitude at each staging site were excluded (Additional file 1: Appendix S1).

Normalized Difference Vegetation Index (NDVI) was used to estimate vegetation conditions [42]. NDVI was obtained from the MODIS satellite product MOD13C1 [43]. Mean NDVI within a radius of 50 km for each wintering site were extracted with the adehabitat R package [44].

Data were pooled for all analyses because t-tests revealed no differences between the two years in average latitude (*p* = 0.44), longitude (*p* = 0.79) or NDVI (*p* = 0.23).

The western ‘detour’ between the staging sites before and after the Sahara coincided with Equinox. We estimated average westernmost latitude projecting from the mean position of the last European staging sites assuming a speed of 300 km/day (daily migration speed of willow warblers ringed in Denmark [45]).

Longitudinal spread of birds in winter was estimated using the loxodromic distance between longitude of the centre of mass for all individuals and the latitude and longitude of the centre of mass for each individual in five-day intervals in R using SDMTools [46] and geosphere [47].

We correlated arrival date, body mass, wing length and NDVI with longitude to evaluate causes of winter spread using Pearson’s *r* (Note that a weak relationship with arrival date is expected because of extra travel time). We tested for consistent north-south or east-west directional changes and direction of change in NDVI between consecutive winter sites using Sign tests. Lastly, we investigated trends over time in NDVI within sites using Pearson’s *r*. Potential effects of variation in longitudinal distribution of NDVI in earlier years on termination of migration were investigated by correlating site-specific NDVI among the last three winters before capture.

## Results

**Fig. 1:**
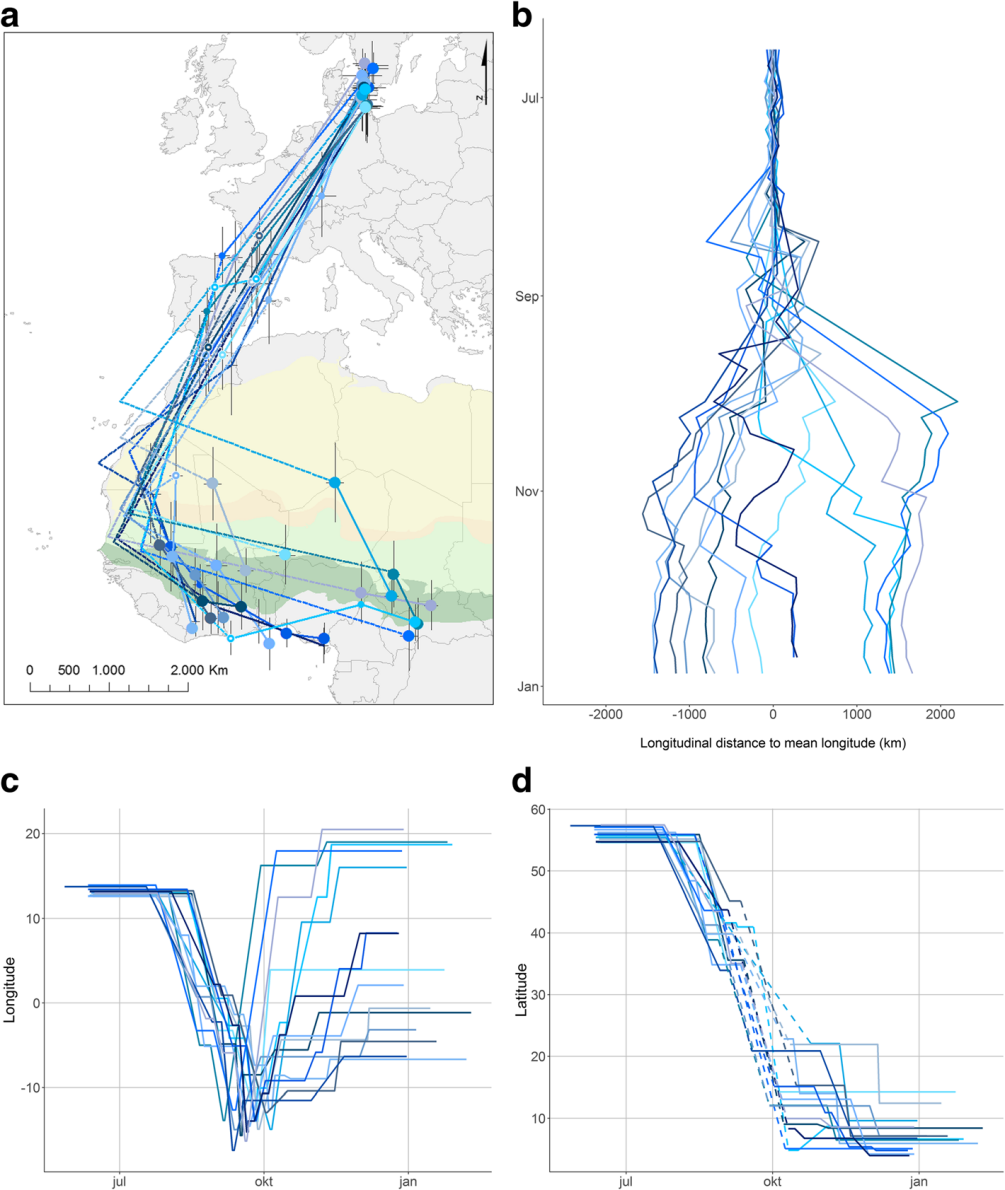
Migration of willow warblers from breeding to wintering grounds (individuals represented by different colours). (**a**) Migration routes and staging sites. Climatic zones are adjusted from Arbonnier [57]. (**b**) Individual longitudinal distances to overall mean. (**c**) Mean longitude and (**d**) latitude with time for stationary periods. Western detours are shown as mean longitude of the three westernmost positions and the latitude three-quarter distance (arbitrarily chosen) between last European and first winter staging sites. Positions during equinox are excluded (represented by dashed lines connecting stationary periods). In (**a**), standard deviation of longitude and latitude, respectively, of positions during each stopover estimated using Hill-Ekström calibration are indicated (potential bias in position estimates from calibration method is not included; Additional file 1: Appendix S2–4)

All willow warblers migrated via the Iberian Peninsula to winter in West and Central Africa from the Sahel to the tropical zone (*n* = 15, Fig. 1). Birds departed the breeding area from late July and arrived in winter grounds from late September (Table 1). Most birds staged at one or more sites before and several after crossing the Sahara (Table 1). Timing of breeding departure and within-winter movements were equally variable (df = 2, F = 1.23, *p* = 0.3; Levene’s test; Fig. 1c,d).

**Table 1:** Summary of migration timing and staging

Migration event	Mean (±SD)	Median	Min	Max
Departure breeding	03 Aug	01 Aug	18 Jul	17 Aug
Arrival winter	10 Oct	09 Oct	18 Sep	13 Nov
Migration Duration	69 ± 14 days	70 days	50 days	92 days
Number of staging sites (of 5 to 12 days duration)	1.7 ± 1.3	1	0	5
Duration of staging before Sahara	13.6 ± 7 days	10 days	7 days	34 days
Duration of staging after Sahara	4.3 ± 6.9 days	0 days	0 days	21 days

**Fig. 2:**
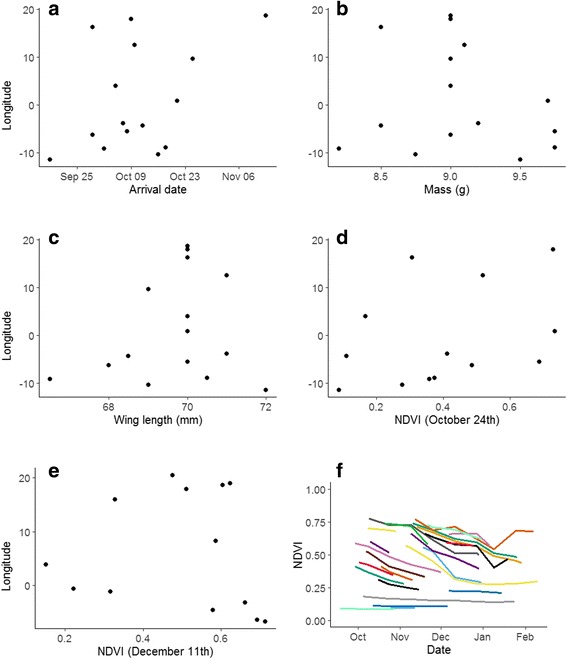
Potential causes of spread in wintering longitudes. (**a**) Arrival timing to wintering grounds, (**b**) body mass, (**c**) wing length, and (**d**) NDVI at first and (**e**) second winter site. (**f**) Temporal vegetation changes at wintering sites (change in NDVI from first to second winter site indicated by gaps)

Migration from Denmark to the westernmost Sahel was along a narrow front (Fig. 1a). The birds detoured considerably toward southwest apparently along the coast, moving east/southeast after reaching their westernmost position at −14.4° ± 1.4° longitude, presumably when reaching the Sahel (average latitude 11.7°). After turning east, individual variation in termination of migration was large (Fig. 1a) with eastward movement of on average 15.7° ± 11.0° (first winter site longitudes −11.5° to 19.7°). Consequently, winter sites were widely spread longitudinally with >3000 km between extremes (Fig. 1b). This was apparently not related to timing of arrival on the wintering grounds (Fig. 2a; longitude and winter arrival: *r* = 0.41, *p* = 0.13), biometrics (Fig. 2b-c; longitude and body mass: *r* = −0.18, *p* > 0.52; wing length: *r* = 0.18, *p* > 0.50) or NDVI (Fig. 2d-e; first winter site: *r* = 0.03, *p* = 0.94; second winter site: *r* = 0.24, *p* = 0.51). Moreover, NDVI in earlier years appeared not to determine termination (*r* = 0.99 for site-specific NDVI among winters).

Most birds used at least two wintering sites, moving between late October and mid-December (Fig. 1a–d). The second site was south (*p* = 0.0005) and east (*p* = 0.0005) of the first. Moving to another site resulted in increased vegetation greenness (Fig. 2f; *p* = 0.02). Greenness generally decreased during an individual’s stay at each site (Fig. 2f; NDVI slope = −0.0025 ± 0.0002 day^−1^) until mid-January. From mid-January greenness increased for three of six stationary individuals.

## Discussion

Our results highlight considerable individual variation in spatiotemporal migration schedules of a small long-distance migrant, the willow warbler. Despite migration to sub-Saharan Africa on a narrow front, we found individuals dispersing widely in winter (>3000 km) by variable termination of migration, resulting in low connectivity (individuals spread over the known wintering area of the subspecies) and considerable difference in migration length among individuals. Vegetation conditions generally declined at each wintering site but within-winter movements toward southeast increased vegetation greenness.

The southwest autumn migration route from Europe to West Africa which turns anticlockwise after reaching the Sahel and with stopovers before and after the desert crossing is likely a common migration pattern for West European birds [15, 17–19] and following the coast rather than crossing the Sahara directly is presumably safer. The willow warblers wintered in the Savannah zone, which is well documented as the main wintering area [28]. However, the easternmost sites were further east of the range considered for this subspecies [29] though separation of subspecies on the wintering grounds is not well known.

The very large longitudinal spread is surprising and in contrast to the high connectivity and limited west-east movement seen in for example common nightingale *Luscinia megarhynchus* [9]. However, similar variable termination of migration also caused large within-population variation in wintering longitudes in some swift populations [48] but not others [49]. Northern and eastern willow warblers migrate via the eastern Mediterranean [31] but it is unknown whether a similar spread westwards into western birds’ ranges occurs in winter. Such spread is assumed in lesser whitethroats *Sylvia curruca* that cross the eastern Mediterranean [50], turning westwards after crossing the Sahara, to winter in the Sahel zone from Ethiopia to Senegal [28].

We did not find support for a temporal progression in settling patterns that would suggest ‘filling up’ of arriving migrants as cause. Nor did we find a relationship between body size and longitude that could be expected if a dominance hierarchy or flying capability were determinant. Furthermore, the similar seasonal vegetation conditions in eastern and western wintering sites indicate that travelling further did not generally result in improved habitat conditions. Potentially, birds could be wintering where they successfully wintered in their first year as suggested by Cresswell [35]. However, we found no support for termination of migration resulting from variation among years in geographic distribution of favourable vegetation conditions, which could potentially influence survival, nor did we find a difference between longitudes of tracked birds in the two years with tracks. Experience-dependent migration routes are known in other species (for example Eurasian honey buzzards *Pernis apivorus* [51, 52]) and because we only tracked experienced adults, birds on their first migration could potentially use a more direct route to the winter grounds without detouring along the coast in which case the spread would be a result of spread in innate migration directions [35]. However, ring-recoveries indicate a general southwest migration direction [45] consistent with the route documented here and indicating that this route is also followed by first-time migrants. Furthermore, such differences between age classes are presumably a result of social learning in larger, diurnally migrating species [51, 52] which is unlikely to happen in the solitary migrating willow warblers.

Migrants are often considered generalists [53] and, compared to for example the common nightingale with high connectivity [9], the willow warbler is associated with a larger variety of more open habitats. Possibly, the spread of individuals could be attributed to generalist species [54] or species foraging in widely distributed open areas. Willow warbler populations appear to be in decline throughout Europe [55]. The low connectivity is consistent with an overall decline associated to factors operating outside the breeding season combined with pronounced regional differences in trends related to breeding area conditions [56]. Itinerant behaviour has also been reported in great reed warblers *Acrocephalus arundinaceus* [27] and common nightingales [26] but not in common redstarts *Phoenicurus phoenicurus* [15] and pied flycatchers *Ficedula hypoleuca* [11]. Movement during winter resulted in improved vegetation conditions when conditions deteriorate in northern Savannah and Sahel areas, as found in Montagu’s harriers [23]. Because spring migration could not be tracked, it remains unknown whether the autumn route is retraced or a more direct route is chosen (as indicated by more easterly ring recoveries in the Mediterranean in spring than autumn; [56]). Such tracking is dependent on further development of device capabilities.

## Conclusion

Willow warblers migrate from North Europe to sub-Saharan Africa on a narrow front, but disperse widely over the Savannah zone after reaching the Sahel. During winter, birds moved southward tracking greenness in vegetation. Wintering sites were spread out with >3000 km between the most eastern and most western individual resulting in low connectivity. The low connectivity in this small, long distance generalist migrant wintering in the Savannah zone may be related to the species being a generalist and is consistent with factors operating outside the breeding area being responsible for the species’ decline.

## Additional files

Additional file 1: Appendix. (DOCX 481 kb)

Additional file 2: Data. (XLSX 312 kb)
