# Supplementary material for: No apparent gain from continuing migration for more than 3000 kilometres: willow warblers breeding in Denmark winter across the entire northern Savannah as revealed by geolocators
Source: Mov Ecol. 2017 Aug 30;5:17. doi: 10.1186/s40462-017-0109-x (PMC5576281; doi:10.1186/s40462-017-0109-x)
Supplement: Supplementary file 1 — Appendix. (DOCX 481 kb) [file 40462_2017_109_MOESM1_ESM.docx]

**Table S1.** The table is showing timing and mean position for the staging sites along with the westernmost position for each individual. Staging sites shown on the map (figure 1a) is indicated in **bold**.

| **Bird ID** | **Stationary Area** | **Start Date** | **End Date** | **Mean latitude** | **SD latitude** | **Mean longitude** | **SD longitude** |
| --- | --- | --- | --- | --- | --- | --- | --- |
| **L898** | **Breeding** | 13/06/2014 | 14/08/2014 | 54.60 | 2.26 | 13.01 | 1.66 |
|  | **Staging** | 02/09/2014 | 11/09/2014 | 34.82 | 3.27 | -3.25 | 0.61 |
|  | Westernmost points | 26/09/2014 | 27/09/2014 |  |  | -13.98 | 0.39 |
|  | **Winter** | 05/10/2014 | 24/01/2015 | 14.21 | 3.31 | 3.91 | 1.13 |
| **L904** | **Breeding** | 13/06/2014 | 13/08/2014 | 55.75 | 1.91 | 13.01 | 1.45 |
|  | **Staging** | 31/08/2014 | 08/09/2014 | 41.63 | 2.59 | 0.54 | 0.62 |
|  | **Staging** | 09/09/2014 | 19/09/2014 | 40.98 | 2.88 | -4.17 | 0.56 |
|  | Westernmost points | 24/09/2014 | 26/09/2014 |  |  | -12.55 | 0.42 |
|  | Staging | 27/09/2014 | 03/10/2014 |  |  | -10.06 | 0.45 |
|  | **Staging** | 11/10/2014 | 17/10/2014 | 4.81 | 1.96 | -2.31 | 0.28 |
|  | **Staging** | 04/11/2014 | 10/11/2014 | 8.68 | 1.24 | 12.52 | 0.41 |
|  | **Winter** | 13/11/2014 | 29/01/2015 | 6.62 | 2.12 | 18.68 | 0.53 |
| **L906** | **Breeding** | 12/06/2014 | 26/07/2014 | 55.44 | 2.40 | 12.66 | 1.71 |
|  | Staging | 08/09/2014 | 16/09/2014 |  |  | -2.68 | 1.72 |
|  | Westernmost points | 05/10/2014 | 06/10/2014 |  |  | -14.91 | 1.55 |
|  | **Winter** | 25/10/2014 | 12/11/2014 | 22.05 | 4.22 | 9.55 | 1.03 |
|  | **Winter** | 18/11/2014 | 31/12/2014 | 9.63 | 2.18 | 15.99 | 0.81 |
| **L935** | **Breeding** | 15/06/2014 | 01/08/2014 | 54.68 | 1.86 | 13.08 | 1.32 |
|  | **Staging** | 21/08/2014 | 28/08/2014 | 38.89 | 1.99 | -4.99 | 0.70 |
|  | Westernmost points | 05/09/2014 | 06/09/2014 |  |  | -13.86 | 1.51 |
|  | **Winter** | 29/09/2014 | 04/11/2014 | 12.07 | 3.76 | 16.21 | 0.68 |
|  | **Winter** | 10/11/2014 | 26/01/2015 | 6.47 | 2.91 | 18.98 | 0.64 |
| **L961** | **Breeding** | 14/06/2014 | 16/08/2014 | 55.90 | 1.71 | 12.89 | 1.39 |
|  | **Staging** | 03/09/2014 | 13/09/2014 | 35.60 | 4.26 | -4.86 | 0.75 |
|  | Westernmost points | 16/09/2014 | 17/09/2014 |  |  | -15.63 | 1.12 |
|  | staging | 18/09/2014 | 29/09/2014 |  |  | -8.49 | 0.47 |
|  | **Winter** | 08/10/2014 | 29/10/2014 | 9.04 | 3.31 | -5.55 | 0.59 |
|  | **Winter** | 05/11/2014 | 10/02/2015 | 8.40 | 2.62 | -1.14 | 0.67 |
| **L965** | **Breeding** | 14/06/2014 | 16/08/2014 | 55.16 | 2.08 | 12.60 | 1.73 |
|  | **Staging** | 01/09/2014 | 07/09/2014 | 41.36 | 2.97 | 0.06 | 0.54 |
|  | Staging | 14/09/2014 | 22/09/2014 |  |  | -2.72 | 0.59 |
|  | Westernmost points | 27/09/2014 | 28/09/2014 |  |  | -14.88 | 0.56 |
|  | **Winter** | 12/10/2014 | 06/12/2014 | 21.91 | 3.90 | -4.35 | 1.00 |
|  | **Winter** | 07/12/2014 | 15/01/2015 | 12.45 | 2.49 | -0.63 | 0.92 |
| **L967** | **Breeding** | 13/06/2014 | 01/08/2014 | 56.23 | 1.37 | 12.92 | 1.20 |
|  | **Staging** | 19/08/2014 | 06/09/2014 | 39.82 | 3.91 | 1.98 | 0.85 |
|  | Staging | 12/09/2014 | 18/09/2014 |  |  | -1.36 | 0.54 |
|  | Staging | 25/09/2014 | 02/10/2014 |  |  | -7.35 | 0.68 |
|  | Westernmost points | 04/10/2014 | 06/10/2014 |  |  | -12.48 | 0.29 |
|  | **Staging** | 08/10/2014 | 14/10/2014 | 22.82 | 4.93 | -8.56 | 0.76 |
|  | **Winter** | 18/10/2014 | 10/11/2014 | 14.00 | 2.82 | -8.95 | 0.65 |
|  | **Winter** | 12/11/2014 | 07/02/2015 | 5.91 | 2.35 | -6.66 | 0.61 |
| **L974** | **Breeding** | 13/06/2014 | 21/07/2014 | 56.05 | 1.27 | 12.67 | 1.08 |
|  | **Staging** | 19/08/2014 | 28/08/2014 | 41.28 | 3.55 | 0.72 | 1.71 |
|  | Westernmost points | 12/09/2014 | 14/09/2014 |  |  | -14.94 | 0.81 |
|  | **Winter** | 29/09/2014 | 01/12/2014 | 12.01 | 2.98 | -6.36 | 0.48 |
|  | **Winter** | 07/12/2014 | 06/01/2015 | 7.13 | 1.22 | -3.17 | 0.54 |
| **L981** | **Breeding** | 12/06/2014 | 17/08/2014 | 54.77 | 2.10 | 13.22 | 1.59 |
|  | **Staging** | 04/09/2014 | 11/09/2014 | 45.19 | 2.30 | 0.91 | 0.60 |
|  | Westernmost points | 02/10/2014 | 03/10/2014 |  |  | -12.94 | 1.56 |
|  | **Winter** | 16/10/2014 | 15/11/2014 | 15.27 | 2.69 | -10.40 | 0.76 |
|  | **Winter** | 19/11/2014 | 19/01/2015 | 7.12 | 1.87 | -4.56 | 0.70 |
| **U023** | **Breeding** | 11/06/2015 | 24/07/2015 | 56.69 | 1.63 | 12.62 | 2.25 |
|  | **Staging** | 09/08/2015 | 14/08/2015 | 48.39 | 3.23 | 8.01 | 1.69 |
|  | **Staging** | 24/08/2015 | 11/09/2015 | 34.84 | 4.28 | -5.13 | 1.72 |
|  | West detour | 21/09/2015 | 22/09/2015 |  |  | -14.53 | 0.61 |
|  | **Winter** | 07/10/2015 | 19/11/2015 | 13.08 | 4.59 | -3.90 | 1.20 |
|  | **Winter** | 30/11/2015 | 29/12/2015 | 4.18 | 3.01 | 2.10 | 0.48 |
| **U024** | **Breeding** | 11/06/2015 | 24/07/2015 | 57.12 | 0.96 | 13.89 | 1.73 |
|  | **Staging** | 19/08/2015 | 30/08/2015 | 43.65 | 2.31 | -3.27 | 0.87 |
|  | West detour | 11/09/2015 | 13/09/2015 |  |  | -12.62 | 1.24 |
|  | **Winter** | 09/10/2015 | 28/12/2015 | 5.07 | 3.97 | 17.93 | 1.02 |
| **U031** | **Breeding** | 11/06/2015 | 13/08/2015 | 55.92 | 1.57 | 13.39 | 1.89 |
|  | Staging | 11/09/2015 | 20/09/2015 |  |  | -5.08 | 0.77 |
|  | West detour | 25/09/2015 | 26/09/2015 |  |  | -13.87 | 0.19 |
|  | **Winter** | 02/10/2015 | 27/10/2015 | 15.09 | 4.94 | -9.18 | 0.84 |
|  | **Staging** | 03/11/2015 | 07/11/2015 | 10.91 | 2.26 | -5.84 | 0.78 |
|  | **Winter** | 18/11/2015 | 02/12/2015 | 5.37 | 1.64 | 4.04 | 0.43 |
|  | **Winter** | 05/12/2015 | 25/12/2015 | 4.77 | 1.84 | 8.27 | 0.54 |
| **U032** | **Breeding** | 27/05/2015 | 18/07/2015 | 57.33 | 1.44 | 13.72 | 1.88 |
|  | **Staging** | 29/08/2015 | 04/09/2015 | 33.93 | 4.94 | -2.25 | 0.67 |
|  | West detour | 11/09/2015 | 12/09/2015 |  |  | -17.39 | 0.49 |
|  | **Winter** | 18/09/2015 | 31/10/2015 | 20.86 | 5.31 | -11.50 | 1.27 |
|  | **Winter** | 21/11/2015 | 31/12/2015 | 6.74 | 1.75 | -6.32 | 0.53 |
| **U033** | **Breeding** | 12/06/2015 | 03/08/2015 | 54.71 | 2.49 | 13.12 | 1.95 |
|  | **Staging** | 30/08/2015 | 03/09/2015 | 43.74 | 2.20 | 2.19 | 1.05 |
|  | Staging | 11/09/2015 | 15/09/2015 |  |  | -2.63 | 0.62 |
|  | West detour | 20/09/2015 | 21/09/2015 |  |  | -15.23 | 0.82 |
|  | Staging | 21/09/2015 | 25/09/2015 |  |  | -13.92 | 0.92 |
|  | Staging | 30/09/2015 | 04/10/2015 |  |  | -10.68 | 0.57 |
|  | **Staging** | 11/10/2015 | 15/10/2015 | 8.31 | 2.96 | -3.77 | 0.38 |
|  | **Winter** | 21/10/2015 | 21/11/2015 | 6.74 | 2.51 | 0.81 | 0.62 |
|  | **Winter** | 01/12/2015 | 26/12/2015 | 3.96 | 2.03 | 8.23 | 0.57 |
| **U037** | **Breeding** | 15/06/2015 | 25/07/2015 | 57.44 | 0.61 | 12.94 | 1.37 |
|  | **Staging** | 25/08/2015 | 30/08/2015 | 43.48 | 2.94 | -1.86 | 0.86 |
|  | **Staging** | 05/09/2015 | 12/09/2015 | 35.03 | 3.52 | -5.90 | 0.52 |
|  | West detour | 19/09/2015 | 20/09/2015 |  |  | -16.29 | 0.82 |
|  | **Winter** | 10/10/2015 | 31/10/2015 | 9.96 | 3.53 | 12.50 | 0.58 |
|  | **Winter** | 07/11/2015 | 29/12/2015 | 8.57 | 2.84 | 20.47 | 0.77 |

**Figure S1.** Migration of willow warblers from breeding to wintering grounds (individuals represented by different colours) estimated with breeding area calibration. (a) Migration routes and staging sites. Climatic zones are adjusted from Arbonnier [27]. (b) Individual longitudinal distances to overall mean. (c) Mean longitude and (d) latitude with time for stationary periods. Western detours are shown as mean longitude of the three westernmost positions and the latitude three-quarter distance (arbitrarily chosen) between last European and first winter staging sites. Positions during equinox are excluded (represented by dashed lines connecting stationary periods).

**
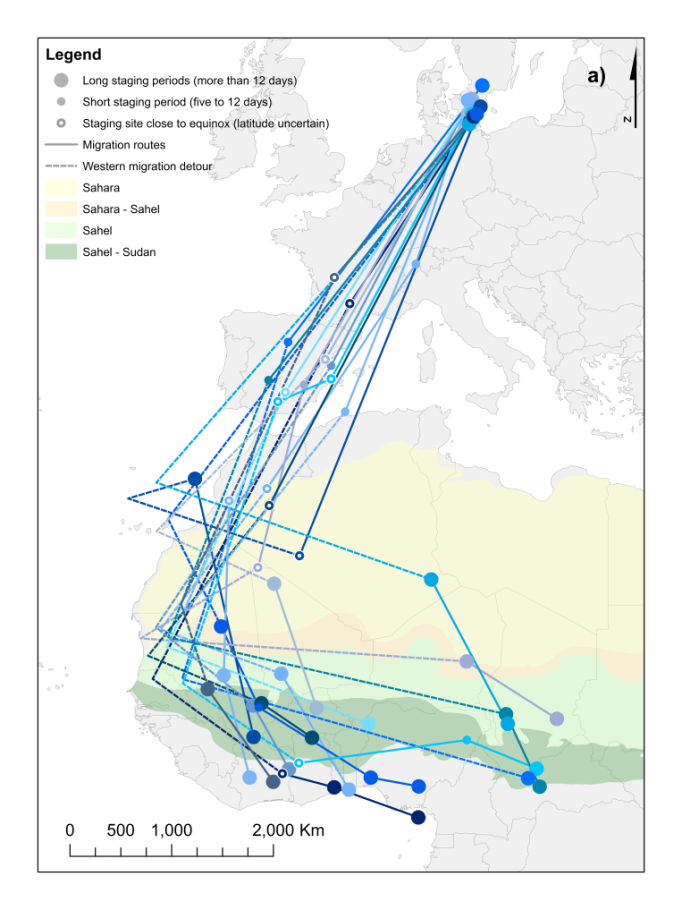

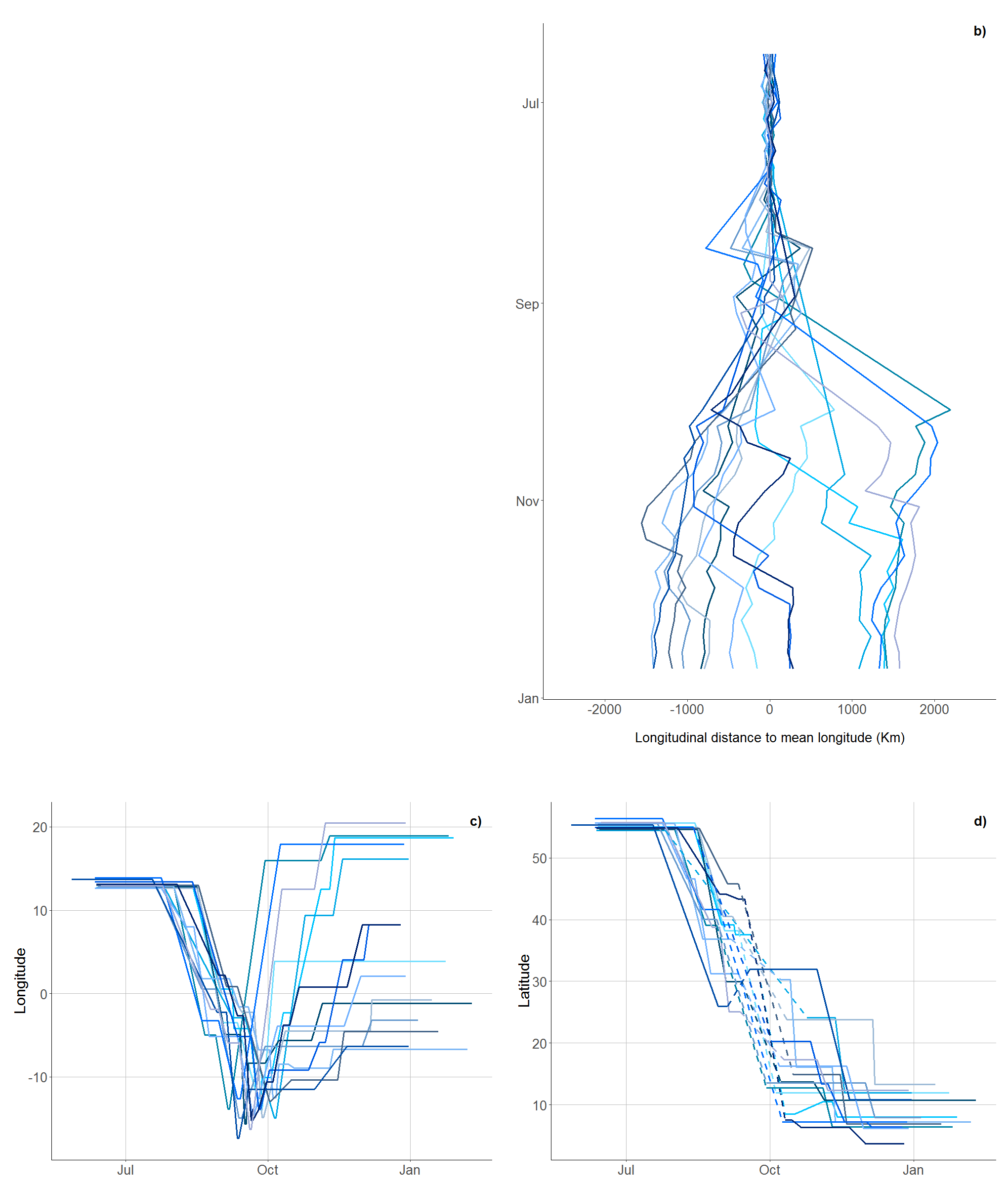
**

**Figure S2.** Potential causes of spread in wintering longitudes using breeding area calibrated positions. (a) Arrival timing to wintering grounds, (b) body mass, (c) wing length, and (d) NDVI at first and (e) second winter site. (f) Temporal vegetation changes at wintering sites (change in NDVI from first to second winter site indicated by gaps).

**
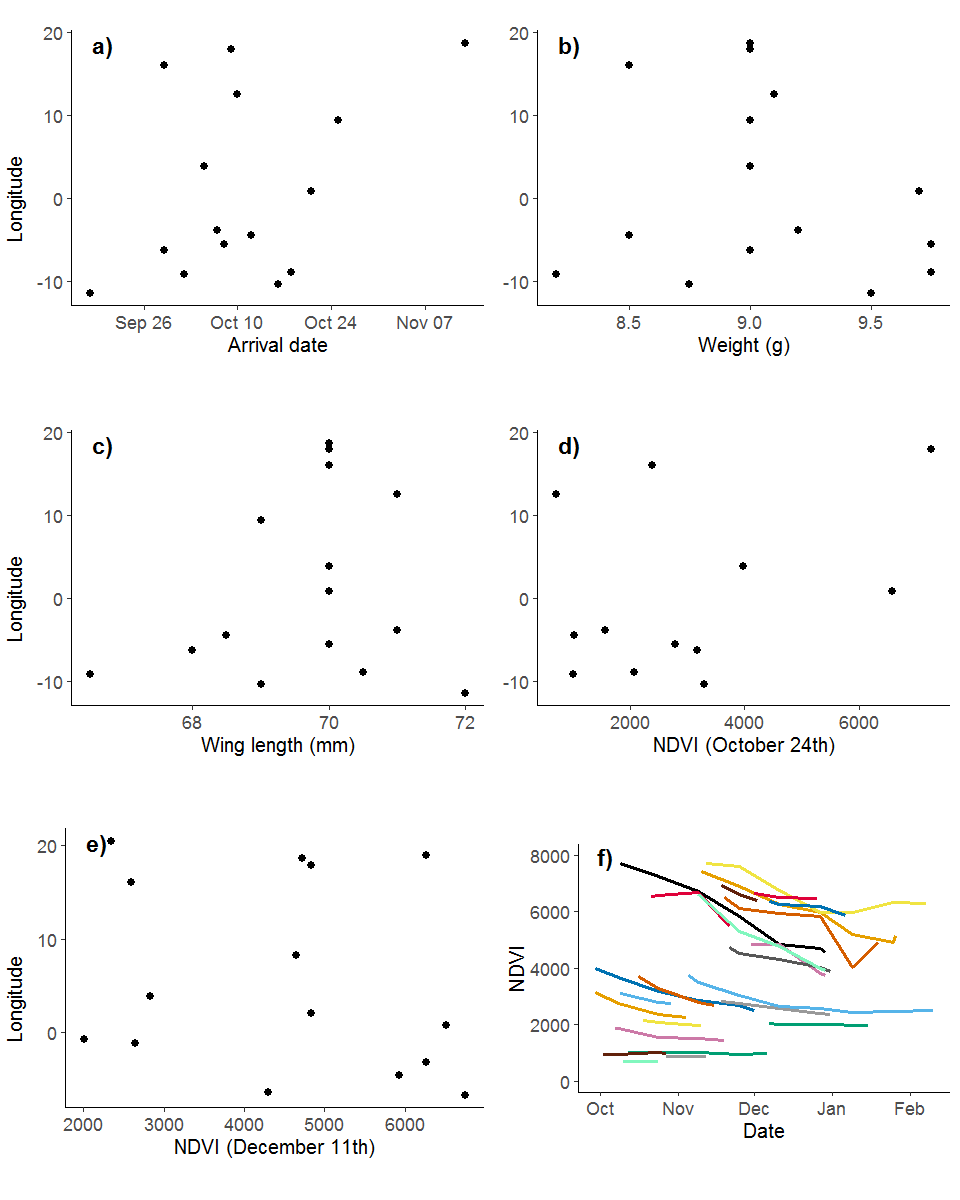
**

**Table S2.** The table is showing timing and mean position for the staging sites estimated with breeding area calibration. Staging sites shown on the map (figure S1a) is indicated in **bold**.

| **Bird ID** | **Stationary Area** | **Start Date** | **End Date** | **Mean latitude** | **SD latitude** | **Mean longitude** | **SD longitude** |
| --- | --- | --- | --- | --- | --- | --- | --- |
| **L898** | **Breeding** | 13/06/2014 | 14/08/2014 | 55.70 | 2.19 | 13.01 | 1.75 |
|  | **Staging** | 02/09/2014 | 11/09/2014 | 38.23 | 3.32 | -3.51 | 0.71 |
|  | Westernmost points | 26/09/2014 | 27/09/2014 |  |  | -13.98 | 0.39 |
|  | **Winter** | 05/10/2014 | 24/01/2015 | 11.94 | 3.33 | 3.85 | 1.25 |
| **L904** | **Breeding** | 13/06/2014 | 13/08/2014 | 55.01 | 1.99 | 13.00 | 1.45 |
|  | **Staging** | 31/08/2014 | 08/09/2014 | 39.16 | 2.90 | 0.54 | 0.62 |
|  | **Staging** | 09/09/2014 | 19/09/2014 | 37.58 | 3.00 | -4.17 | 0.56 |
|  | Westernmost points | 24/09/2014 | 26/09/2014 |  |  | -12.55 | 0.42 |
|  | Staging | 27/09/2014 | 03/10/2014 |  |  | -10.06 | 0.45 |
|  | **Staging** | 11/10/2014 | 17/10/2014 | 8.50 | 2.25 | -2.31 | 0.28 |
|  | **Staging** | 04/11/2014 | 10/11/2014 | 10.51 | 1.23 | 12.52 | 0.41 |
|  | **Winter** | 13/11/2014 | 29/01/2015 | 8.01 | 2.14 | 18.68 | 0.53 |
| **L906** | **Breeding** | 12/06/2014 | 26/07/2014 | 54.45 | 2.52 | 12.72 | 1.88 |
|  | Staging | 08/09/2014 | 16/09/2014 |  |  | -2.87 | 4.23 |
|  | Westernmost points | 05/10/2014 | 06/10/2014 |  |  | -14.91 | 1.55 |
|  | **Winter** | 25/10/2014 | 12/11/2014 | 24.09 | 4.08 | 9.37 | 1.22 |
|  | **Winter** | 18/11/2014 | 31/12/2014 | 11.93 | 2.61 | 16.13 | 1.05 |
| **L935** | **Breeding** | 15/06/2014 | 01/08/2014 | 54.73 | 1.86 | 13.05 | 1.32 |
|  | **Staging** | 21/08/2014 | 28/08/2014 | 39.07 | 1.98 | -4.99 | 0.70 |
|  | Westernmost points | 05/09/2014 | 06/09/2014 |  |  | -13.86 | 1.51 |
|  | **Winter** | 29/09/2014 | 04/11/2014 | 12.76 | 4.11 | 15.98 | 0.85 |
|  | **Winter** | 10/11/2014 | 26/01/2015 | 6.42 | 3.04 | 18.95 | 0.67 |
| **L961** | **Breeding** | 14/06/2014 | 16/08/2014 | 54.59 | 1.98 | 12.80 | 1.42 |
|  | **Staging** | 03/09/2014 | 13/09/2014 | 29.93 | 4.16 | -4.93 | 0.75 |
|  | Westernmost points | 16/09/2014 | 17/09/2014 |  |  | -15.63 | 1.12 |
|  | staging | 18/09/2014 | 29/09/2014 |  |  | -8.34 | 1.36 |
|  | **Winter** | 08/10/2014 | 29/10/2014 | 13.71 | 3.50 | -5.62 | 0.57 |
|  | **Winter** | 05/11/2014 | 10/02/2015 | 10.72 | 2.63 | -1.15 | 0.69 |
| **L965** | **Breeding** | 14/06/2014 | 16/08/2014 | 54.96 | 1.99 | 12.62 | 1.70 |
|  | **Staging** | 01/09/2014 | 07/09/2014 | 40.49 | 2.43 | 0.02 | 0.52 |
|  | Staging | 14/09/2014 | 22/09/2014 |  |  | -2.27 | 43.99 |
|  | Westernmost points | 27/09/2014 | 28/09/2014 |  |  | -14.88 | 0.56 |
|  | **Winter** | 12/10/2014 | 06/12/2014 | 23.75 | 3.71 | -4.49 | 1.12 |
|  | **Winter** | 07/12/2014 | 15/01/2015 | 13.29 | 2.78 | -0.75 | 1.03 |
| **L967** | **Breeding** | 13/06/2014 | 01/08/2014 | 55.56 | 1.40 | 12.87 | 1.23 |
|  | **Staging** | 19/08/2014 | 06/09/2014 | 36.87 | 4.47 | 1.77 | 1.14 |
|  | Staging | 12/09/2014 | 18/09/2014 |  |  | -1.58 | 0.74 |
|  | Staging | 25/09/2014 | 02/10/2014 |  |  | -6.76 | 1.06 |
|  | Westernmost points | 04/10/2014 | 06/10/2014 |  |  | -12.48 | 0.29 |
|  | Staging | 08/10/2014 | 14/10/2014 | 30.29 | 5.81 | -8.48 | 0.78 |
|  | **Winter** | 18/10/2014 | 10/11/2014 | 16.10 | 2.92 | -8.95 | 0.65 |
|  | **Winter** | 12/11/2014 | 07/02/2015 | 7.23 | 2.35 | -6.66 | 0.61 |
| **L974** | **Breeding** | 13/06/2014 | 21/07/2014 | 55.59 | 1.29 | 12.77 | 1.05 |
|  | **Staging** | 19/08/2014 | 28/08/2014 | 40.06 | 3.63 | 0.54 | 1.71 |
|  | Westernmost points | 12/09/2014 | 14/09/2014 |  |  | -14.94 | 0.81 |
|  | **Winter** | 29/09/2014 | 01/12/2014 | 13.52 | 2.87 | -6.35 | 0.48 |
|  | **Winter** | 07/12/2014 | 06/01/2015 | 7.96 | 1.30 | -3.19 | 0.54 |
| **L981** | **Breeding** | 12/06/2014 | 17/08/2014 | 54.79 | 2.33 | 12.97 | 1.83 |
|  | **Staging** | 04/09/2014 | 11/09/2014 | 45.78 | 2.37 | 0.83 | 0.58 |
|  | Westernmost points | 02/10/2014 | 03/10/2014 |  |  | -12.94 | 1.56 |
|  | **Winter** | 16/10/2014 | 15/11/2014 | 14.93 | 2.98 | -10.36 | 0.88 |
|  | **Winter** | 19/11/2014 | 19/01/2015 | 6.85 | 2.03 | -4.58 | 0.74 |
| **U023** | **Breeding** | 11/06/2015 | 24/07/2015 | 55.66 | 1.67 | 12.62 | 2.25 |
|  | **Staging** | 09/08/2015 | 14/08/2015 | 46.60 | 3.40 | 8.01 | 1.69 |
|  | **Staging** | 24/08/2015 | 11/09/2015 | 31.22 | 16.44 | -5.13 | 1.72 |
|  | West detour | 21/09/2015 | 22/09/2015 |  |  | -14.53 | 0.61 |
|  | **Winter** | 07/10/2015 | 19/11/2015 | 16.25 | 4.78 | -3.88 | 1.19 |
|  | **Winter** | 30/11/2015 | 29/12/2015 | 6.16 | 3.04 | 2.10 | 0.48 |
| **U024** | **Breeding** | 11/06/2015 | 24/07/2015 | 56.39 | 0.97 | 13.88 | 1.74 |
|  | **Staging** | 19/08/2015 | 30/08/2015 | 41.64 | 2.42 | -3.27 | 0.87 |
|  | West detour | 11/09/2015 | 13/09/2015 |  |  | -12.62 | 1.24 |
|  | **Winter** | 09/10/2015 | 28/12/2015 | 7.17 | 4.37 | 17.93 | 1.05 |
| **U031** | **Breeding** | 11/06/2015 | 13/08/2015 | 54.96 | 1.68 | 13.40 | 1.89 |
|  | **Staging** | 11/09/2015 | 20/09/2015 |  |  | -5.16 | 0.75 |
|  | West detour | 25/09/2015 | 26/09/2015 |  |  | -13.87 | 0.19 |
|  | **Winter** | 02/10/2015 | 27/10/2015 | 20.23 | 5.07 | -9.18 | 0.84 |
|  | **Staging** | 03/11/2015 | 07/11/2015 | 13.34 | 2.31 | -5.84 | 0.78 |
|  | **Winter** | 18/11/2015 | 02/12/2015 | 7.22 | 1.68 | 4.04 | 0.43 |
|  | **Winter** | 05/12/2015 | 25/12/2015 | 6.44 | 1.85 | 8.27 | 0.54 |
| **U032** | **Breeding** | 27/05/2015 | 18/07/2015 | 55.34 | 1.54 | 13.72 | 1.88 |
|  | **Staging** | 29/08/2015 | 04/09/2015 | 25.99 | 4.99 | -2.25 | -2.25 |
|  | West detour | 11/09/2015 | 12/09/2015 |  |  | -17.39 | 0.49 |
|  | **Winter** | 18/09/2015 | 31/10/2015 | 31.97 | 4.95 | -11.49 | 1.30 |
|  | **Winter** | 21/11/2015 | 31/12/2015 | 10.78 | 1.85 | -6.32 | 0.53 |
| **U033** | **Breeding** | 12/06/2015 | 03/08/2015 | 54.85 | 2.47 | 13.12 | 1.95 |
|  | **Staging** | 30/08/2015 | 03/09/2015 | 44.14 | 2.18 | 2.19 | 1.05 |
|  | Staging | 11/09/2015 | 15/09/2015 |  |  | -2.63 | 0.62 |
|  | West detour | 20/09/2015 | 21/09/2015 |  |  | -15.23 | 0.82 |
|  | Staging | 21/09/2015 | 25/09/2015 |  |  | -13.92 | 0.92 |
|  | Staging | 30/09/2015 | 04/10/2015 |  |  | -10.61 | 0.55 |
|  | **Staging** | 11/10/2015 | 15/10/2015 | 7.56 | 2.92 | -3.77 | 0.38 |
|  | **Winter** | 21/10/2015 | 21/11/2015 | 6.36 | 2.49 | 0.81 | 0.62 |
|  | **Winter** | 01/12/2015 | 26/12/2015 | 3.70 | 2.02 | 8.23 | 0.57 |
| **U037** | **Breeding** | 15/06/2015 | 25/07/2015 | 55.73 | 0.66 | 12.94 | 1.37 |
|  | **Staging** | 25/08/2015 | 30/08/2015 | 38.75 | 3.30 | -1.86 | 0.86 |
|  | **Staging** | 05/09/2015 | 12/09/2015 | 25.04 | 3.23 | -5.93 | 0.53 |
|  | West detour | 19/09/2015 | 20/09/2015 |  |  | -16.29 | 0.82 |
|  | **Winter** | 10/10/2015 | 31/10/2015 | 17.29 | 3.59 | 12.50 | 0.60 |
|  | **Winter** | 07/11/2015 | 29/12/2015 | 12.36 | 2.97 | 20.47 | 0.77 |
